# The Book World of Medicine and Science

**Published:** 1911-11-11

**Authors:** 


### The Book World of Medicine and Science.

### DREAMS, AND THEIR SIGNIFICANCE.

The World of Dreams. By Havelock Ellis. (London : Constable and Co., Ltd., Leicester Square, W.C. Price 7s. 6d. net.)

Whatever Dr. Ellis writes is bound to be interesting. One of the few medical men who may justifiably claim to possess a literary style, which makes his writing a real pleasure to read, he combines with this a catholicity of interest and a broadmindedness in treating his subject which must of necessity appeal to the cultured reader. The wide knowledge of the matters about which he writes is nowhere so well displayed as in this new book, in which he discourses pleasantly about dreams. It is a hackneyed subject this, but one which at the present time has more than ordinary interest for the medical man, owing to the recent developments in what is known as psycho-analysis. In this domain the work of Freud has been singularly fertile of results, and there has arisen a school that claims to be able to effect important diagnoses by a correct and scientific interpretation of dreams. Such interpretation is not in the nature of occult and cabalistic endeavour to explain dream memories by a series of set formulæ, but is based on certain physiological and pathological facts, which afford presumptive cause for the belief that every dream image is the result of a definite physical condition. A great deal of work yet remains to be done before the premisses of the psycho-analytic school can be accepted in their entirety, but enough has already been accomplished to suggest, at least, that the approach of the problem has been from the right direction. Dr. Ellis has chosen the introspective method in attacking the subject—the method that attracted Maury. He claims that his findings are not the outcome of experiment or of any deliberate concentration of thought on dreaming, but simply the jotting down of experiences—dream experiences—such jottings having been penned immediately on awakening from the dream sleep. In a less methodical and less scientifically accurate writer, the reader would be amply justified in questioning the value of such random notes from memory —admittedly a very uncertain guide in describing the experiences during the dream state. Here, however, such suspicion is unwarranted, at least so far as essentials are concerned. Personally, we do not think that the author has proved that critics like Tannery and Foucault, who deny the capacity of the dreamer to "transcribe" his dreams with sufficient accuracy to enable certain conclusions to be drawn from them, are in the wrong. The argument for and against this would take too long to state in a brief review such as this; for a full discussion of the point we must refer readers to Dr. Ellis' illuminative introduction, which considers the whole question from every aspect.

As a merely interesting and suggestive book, Dr. Ellis' new volume will appeal to everyone who is interested in the subject of dreams. The chapter dealing with dreams of the dead—one of the most absorbing subjects that the psychologist can study—is particularly instructive, since the author relates his personal experiences with commendable restraint and precision. If we remember rightly, Ellis was the first investigator who scientifically interested himself in this variety of dreams. "Every path of this garden of dreamland," he remarks, "might lead to the heart of the universe," and surely on no other path do we want more guiding light, more sign-posts, and a less complicated outline of itinerary than on this. When we consider the diverse routes by means of which different persons reach the same "pscychic dissociation," we are struck by the fact that it is impossible to obtain any real finality in conclusions or deductions until we possess a system of classification for what may be styled, perhaps illogically, introspective phenomena. Such a classification we hope Dr. Ellis may yet give us; in this book he has attempted none, but, as he says, it is merely a preliminary, to be followed, let us hope again, by a future work on more comprehensive and more definite lines. Something, we venture to submit, might be gained by the expert analysis of recorded dreams in literature; a great deal more, perhaps, by the careful record of dream experiences on the lines attempted in this book. But at present the subject is too nebulous and inchoate to permit the average man to have more than a dilettante interest in it. Dr. Ellis' new work will tend to strengthen that interest undoubtedly, but it will not afford the classification which we want. This much, at least, it will teach those of us who are interested in the subject, to attach value to the immediate and conscientious noting-down of dream memories. We commend the practice to every medical man.

---

Infectious Diseases : A Practical Text-book. By Claude Buchanan Ker, M.D., F.R.C.P. Edin. Illustrated with colour plates and photographs. The Oxford Medical Publications. (London : Henry Frowde and Hodder and Stoughton. Crown 4to. Pp. 537. Price 20s. net.)

The success of Dr. Ker's text-book has fully confirmed the favourable opinions expressed on all sides at the time of its publication. We may take it that this success is due to two factors : firstly, to the author's thoroughly practical treatment of the subject; and, secondly, to the convincing tone in which the teaching is set out. One gathers that Dr. Ker has appreciated the difficulties of general practitioners and resident medical officers in dealing with infectious diseases, and has set himself the task of recording the results of his large experience, not only in the natural history of the several diseases, but also in the important minutiæ of treatment and general management of a hospital for infectious diseases. The reader's confidence is gained and held, so that he feels at once that he has here a reliable guide, and one whom he can follow with safety, and with as much success as is practically possible. Details must naturally differ according to the personal equation, and administrative methods are partly dependent on local circumstances, but the point we have no hesitation in emphasising is that Dr. Ker's text-book remains at the present time a thoroughly useful and profitable manual. We need not again particularise the features of this handsome volume; to mention that it is one of the Oxford Medical Publications implies that its letterpress is excellent, and that the plates are beautifully reproduced. All the diseases usually included under the heading of infectious diseases are adequately dealt with, while scarlet fever, diphtheria, and typhoid fever naturally receive very full consideration. To every one connected with an isolation hospital, however small, the last chapter must appeal very strongly, as it deals with the ever-recurring problems of cross-infection, return cases, etc. In future editions this chapter might well be made more important and, with the growing tendency to attach less and less importance to clothes and fomites in conveying infection, may possibly undergo some revision. But as regards the accounts of the several diseases and their treatment, we anticipate that but little room for improvement will be found.

